# *VILIP-1* Downregulation in Non-Small Cell Lung Carcinomas: Mechanisms and Prediction of Survival

**DOI:** 10.1371/journal.pone.0001698

**Published:** 2008-02-27

**Authors:** Jian Fu, Kathryn Fong, Alfonso Bellacosa, Eric Ross, Sinoula Apostolou, Daniel E. Bassi, Fang Jin, Jirong Zhang, Paul Cairns, Inmaculada Ibañez de Caceres, Karl-Heinz Braunewell, Andres J. Klein-Szanto

**Affiliations:** 1 Department of Pathology, Fox Chase Cancer Center, Philadelphia, Pennsylvania, United States of America; 2 Human Genetics Program, Fox Chase Cancer Center, Philadelphia, Pennsylvania, United States of America; 3 Biomathematics and Biostatistics, Fox Chase Cancer Center, Philadelphia, Pennsylvania, United States of America; 4 Bioinformatics Facility, Fox Chase Cancer Center, Philadelphia, Pennsylvania, United States of America; 5 Surgical Oncology, Fox Chase Cancer Center, Philadelphia, Pennsylvania, United States of America; 6 Neuroscience Research Center of the Charité, Faculty of Medicine, Humboldt University, Berlin, Germany; University of Oldenburg, Germany

## Abstract

VILIP-1, a member of the neuronal Ca++ sensor protein family, acts as a tumor suppressor gene in an experimental animal model by inhibiting cell proliferation, adhesion and invasiveness of squamous cell carcinoma cells. Western Blot analysis of human tumor cells showed that VILIP-1 expression was undetectable in several types of human tumor cells, including 11 out of 12 non-small cell lung carcinoma (NSCLC) cell lines. The down-regulation of VILIP-1 was due to loss of *VILIP-1* mRNA transcripts. Rearrangements, large gene deletions or mutations were not found. Hypermethylation of the *VILIP-1* promoter played an important role in gene silencing. In most VILIP-1-silent cells the *VILIP-1* promoter was methylated. *In vitro* methylation of the *VILIP-1* promoter reduced its activity in a promoter-reporter assay. Transcriptional activity of endogenous *VILIP-1* promoter was recovered by treatment with 5′-aza-2′-deoxycytidine (5′-Aza-dC). Trichostatin A (TSA), a histone deacetylase inhibitor, potently induced VILIP-1 expression, indicating that histone deacetylation is an additional mechanism of VILIP-1 silencing. TSA increased histone H3 and H4 acetylation in the region of the *VILIP-1* promoter. Furthermore, statistical analysis of expression and promoter methylation (n = 150 primary NSCLC samples) showed a significant relationship between promoter methylation and protein expression downregulation as well as between survival and decreased or absent VILIP-1 expression in lung cancer tissues (p<0.0001). VILIP-1 expression is silenced by promoter hypermethylation and histone deacetylation in aggressive NSCLC cell lines and primary tumors and its clinical evaluation could have a role as a predictor of short-term survival in lung cancer patients.

## Introduction

Visinin-like protein-1 (VILIP-1), a member of the visinin-recoverin neuronal calcium-sensor protein family, has an important role in regulating cAMP levels, cell signaling and differentiation in central nervous system. VILIP-1 has been implicated in pathological processes of the nervous system such as Alzheimer's disease and Schizophrenia [1], [2]. Our group identified VILIP-1 to be differentially expressed in chemically-induced murine skin cancer cells of high and low invasive ability by differential display, indicating a new function of VILIP-1 in cancer [3], [4]. VILIP-1 was expressed in normal basal epidermal keratinocytes, while its expression was markedly decreased or undetectable in aggressive and invasive squamous cell carcinoma (SCC). Conversely, less aggressive SCCs showed expression of VILIP-1 protein. Ectopic overexpression of VILIP-1 resulted in a cAMP-mediated decrease of *in vivo* and *in vitro* growth and invasiveness of SCC cells [3]. Reduced invasiveness and elevated cAMP levels were accompanied by decreased MMP-9 as well as lowered RhoA activity [4]. Furthermore, enforced expression of VILIP-1 led to inhibition of cell adhesion and migration by down-regulating fibronectin receptors, suggestive of a tumor suppressor function for VILIP-1 [4]. Interestingly, a similar tumor suppressor role for VILIP-1 has been reported recently in two other tumor cell types. Wickborn et al [5] found that VILIP-1 expression was completely lost or significantly reduced in esophageal SCC compared with normal squamous epithelium of the same site. Lower VILIP-1 protein expression was correlated with clinical-pathological features including deeper tumor invasion and increased local lymph node metastases. In another study [6], xenotransplanted neuroblastoma cells in which the expression of the pro-tumorigenic gene *MIF* was suppressed by antisense oligonucleotides a significant reduction in tumor growth together with VILIP-1 upregulation was observed, suggesting that VILIP-1 loss is associated with tumor development.

Lung cancer, the leading cause of cancer-related death in the world, is known to result from tobacco carcinogen-induced abnormalities in several critical genes. Genetic approaches have identified a number of oncogenes and tumor suppressor genes gained or lost in human lung cancers [7]. Recently, epigenetic mechanisms, such as DNA methylation and histone modification, have been identified as contributors to the disease phenotype [8]. Since VILIP-1 is involved in the progression of polycyclic aromatic hydrocarbon-induced experimental skin SCCs, we decided to determine whether genetic and epigenetic changes of this gene in tobacco-associated human non-small cell lung carcinomas (NSCLC) would lead to protein expression alterations and whether these changes could affect clinical outcome.

## Materials and Methods

### Cell lines

Non-small cell lung cancer cell lines (NSCLC) A549, NCI-H522, NCI-H460, NCI-H226, NCI-H520, NCI-H23, Calu1, Calu6 were obtained from American Type Culture Collections (Manassas, VA). HOP62, EKOX, NCI-H322 and HOP92 cells were provided by the Fox Chase Cancer Center Cell Culture Facility and cell lysates of NCI-60 panel of tumor cells were obtained from the Translational Research Facility. A549, NCI-H522, NCI-H460, NCI-H226 were cultured in RPMI 1640 supplemented with 10% fetal bovine serum, 2 mM L-glutamine, penicillin (100 IU/ml) and streptomycin (100 µg/ml). NCI-H520 was cultured with RPMI 1640 medium containing 1.5 g/L sodium bicarbonate, 4.5 g/L glucose, 10 mM HEPES, 1.0 mM sodium pyruvate, 2 mM L-glutamine and 10% fetal bovine serum. Calu1 and Calu6 were cultured with McCoy's 5a medium with 1.5 mM L-glutamine and 10% fetal bovine serum. Primary cultures of normal human bronchial epithelial cell (NHBE) derived from 2 different donor sources (NHBE1 and NHBE2) were obtained from Cambrex (Baltimore, MD) and cultured with a BEGM Bullet kit. All cells were cultured at 37°C in a humid incubator with 5% CO_2_.

### Western blot and Northern blot analyses

Cellular protein and RNA were extracted and analyzed as before [3]. VILIP-1 Western analysis of NCI-60 panel of tumor cells was performed with 25 µg of cell lysate. In all other VILIP-1 Western analyses, 40 µg of cell lysate were used. VILIP-1 protein was detected by blotting with rabbit anti-VILIP1 antibody using a 1∶3000 dilution of the original stock. VILIP-1 full-length cDNA was used as probe in Northern blot analysis.

### Mutation analysis by direct sequencing

Exon fragments of *VILIP-1* containing exon and exon-intron junctions were amplified with the primers listed in supplemental Table S1 from cellular genomic DNA and sequenced with the same sets of primers by the Automated DNA Sequencing Facility at Fox Chase Cancer Center. *VILIP-1* promoter was amplified from the genomic DNA using VPFkpn and VPRbgl primers and sequenced with VP2Kb primers (Supplemental Table S1).

### *In vitro* methylation of VILIP-1 promoter and reporter gene assay

The *VILIP-1* promoter was amplified from the genomic DNA of NHBE cells with VP2kb cloning primers as listed in Table S1 and ligated to pGL4.10[luc2] vector (Promega, Madison, WI). *In vitro* methylation of luciferase reporter plasmid was performed as described [9]. The promoter fragment was excised out of 20 µg pGL4.10VP2kb by digestion with the restriction enzymes BglII and KpnI (New England Biolabs, Bevelry, MA) and gel purified by QIAquick Gel Extraction kit (QIAGENGmbH, Hilden, Germany). Half of the purified promoter fragment was methylated with M. SssI DNA methylase (New England Biolabs, Bevelry, MA) and the other half was incubated in the absence of enzyme as mock methylation. Methylated and mock-methylated fragments were relegated into the vector from which they had been excised.

Cells were transfected by Lipofectamine 2000 (Invitrogen, Carlsbad, CA) using the manufacturer's protocol. Briefly, DNA mixture containing methylated or mock-methylated DNA and 8 ng of pGL4.73, a transfection efficiency control, was diluted in 50 µl of Opti-Mem I medium and mixed with 50 µl of diluted Lipofectamine 2000. 100 µl of DNA-Lipofectamine 2000 complexes were added to each well after 20 min incubation at room temperature and cells were left in the incubator for 24 hr before lysis. Reporter gene activity was measured according to Dual-luciferase reporter 1000 assay system kit (Promega, Madison, WI) by using the luminometer Sirius FB15 (Zylux Corporation, Oak Ridge, TN).

### Bisulfite modification of DNA, methylation-specific PCR (MSP) and bisulfite sequencing

Genomic DNA (1µg) was modified with sodium bisulfite as previously described [10], [11]. Bisulfite modification of DNA results in the conversion of unmethylated cytosines to uracils, whereas methylated cytosines are resistant to modification and remain as cytosines [12].

MSP was performed as follows: PCR reactions comprised 2 µl of sodium bisulfite treated DNA, 0.2 mM of dNTPs, 0.2 µM of forward and reverse primers each, 1× reaction buffer, 0.2 mM of MgCl_2_ and 5 units of Ampli Taq Gold DNA polymerase (Applied Biosystems, Foster city, CA). Methylation and non-methylation specific primers (Table S1) were used to uncover the methylation status of sodium bisulfite modified DNA. PCR program for methylation-specific primers: 95°C for 5 min for the first cycle, followed by 95°C for 35 sec, 57°C for 45 sec, and 72°C for 40 sec (40 cycles), and 72°C for 10 min. Except for the use of 60°C as the annealing temperature, the PCR program for nonmethylation-specific primers was the same. The PCR products were visualized on 2.5% agarose gels.

Bisulfite sequencing of 20 CpG sites on the second CpG island *of VILIP-1* promoter was done by PCR amplification of 2 overlapping fragments, the first of which (named 2ori) covered the first 3 CpGs and the second (named 3ori) covered the other 17 CpGs. PCR reaction was performed with the same condition as MSP (except for annealing that was performed at 55°C). The PCR products were ligated into pCR4-TOPO using the TOPO TA cloning system (Invitrogen, Carlsbad, CA) and transformed into bacteria TOPO10. Plasmid DNA was isolated using the Miniprep kit (QIAGENGmbH, Hilden, Germany). Six to 8 clones were sequenced for each sample.

### 5′-Aza-dC and TSA treatment of cells

5′-aza-2′-deoxycytidine (5′-Aza-dC) and trichostatin A (TSA) were purchased from Sigma-Aldrich (St. Louis, MO) and dissolved in DMSO as stock solution. Cells were seeded at low density one day before 5′-Aza-dC treatment and treated for 5 days continuously at concentrations ranging from 0.001 to 1 µM. Medium containing 5′-Aza-dC or DMSO vehicle control was changed every 24 hours. For TSA treatment, cells were plated at 50–70% confluence and incubated for one day followed by treatment with TSA for 20 hours before harvesting cells.

### Chromatin immunoprecipitation assay

Chromatin immunoprecipitation (CHIP) [13] was performed using the Acetyl-Histone H3/H4 Immunoprecipitation assay kit (Upstate Biotechnology, Lake Placid, NY) following the manufacturer's protocol. Briefly, after 20-hour treatment with TSA, histones were cross-linked to DNA by incubating cells with 1% formaldehyde for 10 min. Cell pellets were resuspended in 200 µl of SDS lysis buffer followed by DNA sonication for a total of 16 times (each time for 20s at 30% of maximal power) by using Sonic Dismembrator 550 (Fisher Scientific, Pittsburgh, PA). Approximately pproximately 1% of the lysate was used as input and immunocomplexes were captured from the rest of lysates with 10 µl anti-acetyl histone H3 or H4 antibody. After the cross-linking was reversed by heating the sample at 65°C for 4 h, DNA was extracted with phenol/chloroform and precipitated with ethanol. PCR reactions were performed by using 1% of immunoprecipitated material and the ChIP primers Table S1). The following PCR program was used: 95°C for 5 min followed by 35 cycles of 95°C for 35 s, 54°C for 45s and 72°C for 40s, and finally 72°C for 10 min.

### Tissues and tissue microarrays (TMA)

Frozen and paraffin embedded tissues from the Department of Pahology at Fox Chase Cancer Center were used for MSP and immunohistochemistry (IHC). Informed consent was obtained from the patients for tissue procurement prior to accrual and their medical records and databases were maintained according to institutional guidelines and in conformance with HIPPA regulations. The overall survival data on all patients were censored on the date of the last follow-up visit or death from causes other than lung cancer. All patient information was derived from a de-identified database approved by the FCCC Institutional Review Board. A total of 10 normal lungs, 10 lung adenocarcinomas and 69 SCCs were analyzed. In addition to these samples, TMAs containing additional cases of NSCLC were used to evaluate VILIP-1 expression by IHC. To construct the TMAs, small core biopsies were taken from representative areas of the donor paraffin-embedded lung cancer blocks and assembled onto a 3–4 mm thick paraffin recipient block. This was done with a tissue arrayer (Beecher Instruments, Silver Spring, MD, USA) Two 1mm diameter cores from the same tumor were arranged side by side onto the recipient block to minimize any heterogeneity during the acquisition of the samples with the arrayer. In addition, normal tissue blocks from liver, kidney, colon, lung, etc were placed in all peripheral columns and rows. This diminishes any eventual border artifact during immunohistochemistry and also serves as topological markings to help orient the user. The TMAs contained 64 cores from 43 different squamous cell carcinomas and 132 cores from 65 different adenocarcinomas of the lung. Five micrometer thick sections were obtained with a standard rotary microtome and one section was stained with hematoxylin-eosin to corroborate the histopathological characteristics of the core specimens.

### Immunohistochemistry and MSP analysis

VILIP-1 immunohistochemistry of tumors and normal lung tissues was performed using sections obtained from the TMAs and from conventional paraffin blocks. VILIP-1 was detected using a rabbit polyclonal antibody as described previously [3].

The immunostain was evaluated semiquantitatively using a modified Allred scoring scale [14] that takes into consideration the intensity of the immunostain on a scale of 0–3+, with 0 representing no detectable stain, 1+ minimal stain, 2+ moderate stain, and 3+ representing intense stain. In addition the percent area stained is also added to the intensity scale using indices from 0 to 5, 0 representing no area stained, 1 representing 0–1% stained area, 2: 1–10%, 3: 10–33%, 4: 33–66% and 5: more than 66% of the area stained. Both intensity and area indices are added resulting in a total scale ranging from 0 to 8. As positive and negative control tissues we used paraffin sections of xenografted lung carcinoma cell lines (NCI-H520 and Calu 1) that were grown in Scid mice for 6 weeks, fixed in buffered formaldehyde and embedded in paraffin.

Genomic DNA was extracted from frozen tissues using QIAamp DNA Mini Kit (QIAGENGmbH, Hilden, Germany) and subjected to bisulfite modification and MSP analysis as described above.

### Statistical Analysis

For the evaluation of VILIP-1 immunostaining in tumors, individual tumors were scored in two TMA cores. Eighty-one of the 108 cases in the TMAs had complete clinical annotation and/or follow-up and were used for statistical test involving clinical staging and survival. Two-sample Wilcoxon tests were used to test for differences in the distribution of VILIP1 values across subgroups (i.e., histology (SCC vs. adeno), high-stage (stages 1 and 2) vs. lower stage (stages 3 and 4), and low grade (grades 1 and 2) vs. high grade (grades 3 and 4)). In order to evaluate disease-free survival and overall survival we used Kaplan Meir curves [15]. A plot of the Kaplan-Meier type estimates the survival function in a series of horizontal steps of declining magnitude which, when the sample is large enough, approaches the true survival function for that population. Cox proportional hazards models were used to assess the significance of the relationship between survival time and VILIP1 alone, and VILIP1 after adjusting for stage, grade and histology. Survival time was defined as the time from surgery to death, or date of last follow-up. Individuals who were alive at last contact were censored for these analyses. All tests were two-sided with a 5% type I error.

The two-sample Wilcoxon procedure was used to test for differences in VILIP1 in patients who lived less then 2 years as compared to long term survivors (≥5 years). A chi-square test was used to assess the significance of the association between a dichotomized measure of VILIP1 and presence of VILIP-1 promoter methylation in lung SCCs.

## Results

### VILIP-1 expression is lost in many human humor cell lines

**Figure 1:**
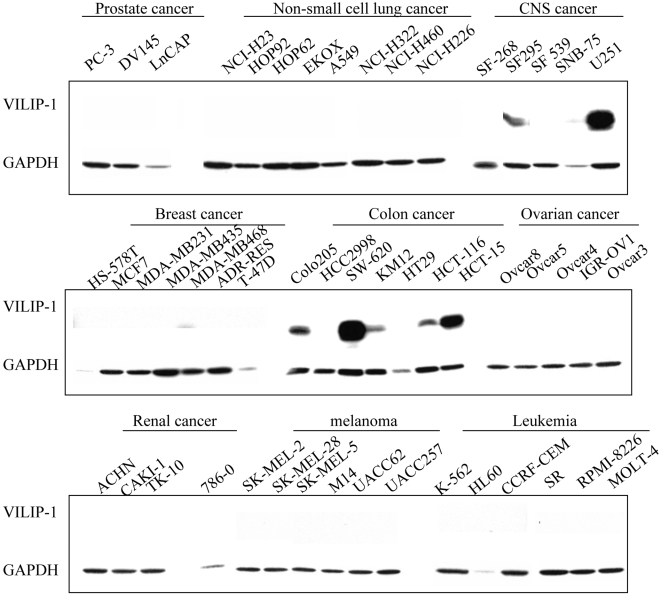
Western analysis of VILIP-1 expression patterns in the NCI-60 panel of tumor cell lines. Note that except for a few tumor cell lines from colon and nervous system, the tumor cell lines did not express VILIP-1.

**Figure 2:**
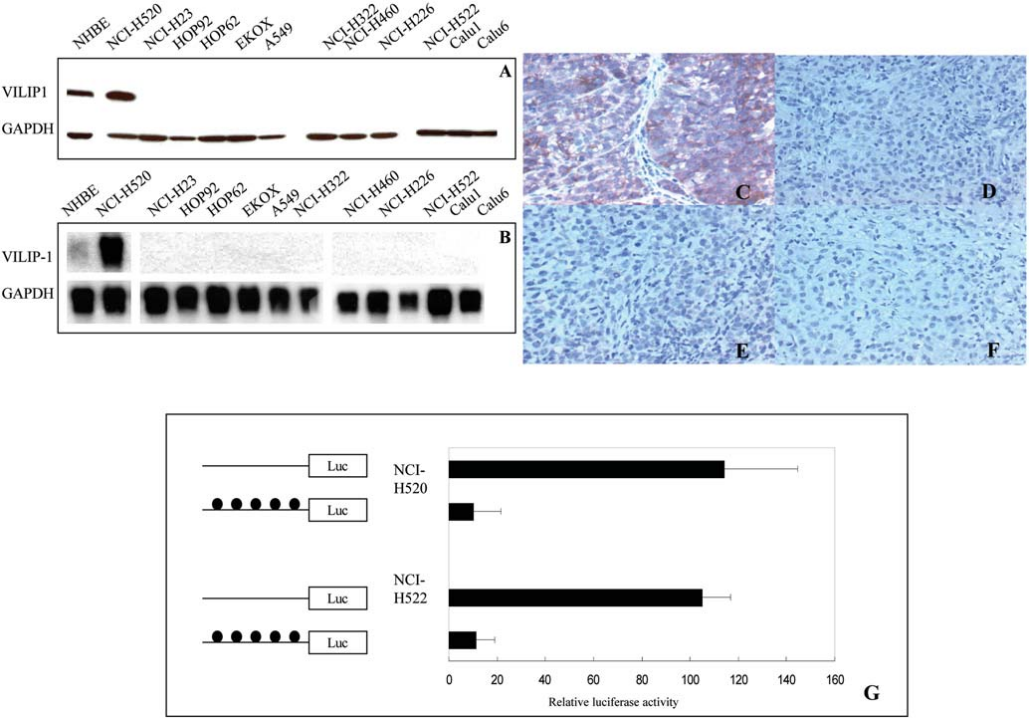
Silencing of the *VILIP-1* gene in human lung cancer cell lines. A. Western blot result from normal human bronchial epithelial (NHBE) cells and 12 NSCLC cell lines. VILIP-1 was identified as 22 kDa. Glyceraldehyde-3 phosphate dehydrogenase (GAPDH) was used as loading control. B. Northern blot of total RNA extracted from NHBE and 12 NSCLC cell lines probed with *VILIP-1* full-length cDNA. Immunohistochemistry of VILIP-1 in subcutaneous tumors derived from the NSCLC cell lines, C: NCI-H520, D: Calu1, E: Calu6, and F: A549. ×100. Effect of methylation on VILIP-1 promoter activity (G). pGL4.10 vector containing *in vitro* methylated (filled circles) or nonmethylated (no circles) VILIP-1 promoter fragment was transfected into NCI-H520 or NCI-H522 cells. Transfection efficiency was normalized to the cotransfected pGL4.73 vector. The data presented as mean±SD (bars) of triplicate experiments.

In order to gain a broad view of VILIP-1 expression patterns in human cancer, we analyzed the NCI-60 panel of cancer cell lysates by Western blot analysis. Except for a few tumor cell lines from central nervous system and colon, VILIP-1 protein was commonly absent in human cancer cell lines, including those derived from prostate, lung, ovarian and renal tumors as well as those from melanoma and leukemia (Figure 1). We focused on lung-derived cells to further examine VILIP-1 protein expression in normal human bronchial epithelial cells (NHBE) and a total of 12 non-small cell lung cancer (NCSLC) cell lines (Figure 2A). Most NSCLC cell lines (11 out of 12) showed low or no VILIP-1 expression. Conversely, VILIP-1 was significantly expressed in NHBE cells. Only one lung cancer cell line, NCI-H520, expressed VILIP-1 protein. To investigate whether VILIP-1 protein down-regulation was caused by silencing of transcription, we performed Northern Blot analysis using total RNA extracted from cells. A single band of 1.6 kb representing the *VILIP-1* mRNA was identified in NCI-H520 and NHBE cells only, indicating that absence of *VILIP-1* RNA transcript led to loss of protein (Figure 2B). Four tumorigenic cell lines (NCI-H520, Calu 1, Calu 6 and A549) were grown *in vivo* as subcutaneous xenografts in Scid mice. Immunohistochemistry of xenografts showed that only NCI-H520-derived tumors exhibited positive VILIP-1 expression (Figure 2C), whereas the other cell line-derived tumors showed no immunostain (Figure 2 D–F).

### Absence of *VILIP-1* gene mutations

Both abnormal genetic and epigenetic events are responsible for development of cancer [7], [8]. We first investigated whether genetic alterations caused VILIP-1 silencing by analyzing the *VILIP-1* gene organization at the genomic DNA level. Southern blot hybridization revealed no truncation, gross deletion or reorganization of the *VILIP-1* gene at the genomic DNA level in VILIP-1 silent cell lines as compared to NCI-H520 and NHBE that expressed VILIP-1 (data not shown). *VILIP-1* is encoded by 4 exons, of which exons 2, 3, and the 5′-terminus of exon 4 contain the coding sequences. To exclude the possibility that nonsense mutations lead to early transcription termination or frameshift mutation resulting in shorter transcripts, we sequenced exons 1, 2, 3, the coding sequence of exon 4 and the exon-intron junctions (Supplemental Table S2). Except for a polymorphism (G to A) detected at the junction between the second exon and intron of NHBE, A549 and Calu1 cell lines, mutations were not found in the VILIP-1 expressing and non-expressing cell lines. Neither deletions nor mutations were found in the 4 exon-intron junctions.

We also explored the possibility that mutations in the *VILIP-1* promoter could lead to aberrant promoter activity thus contributing to downregulation of the *VILIP-1* gene in lung cancer cells. A sequence of approximately 2 kb upstream of the first *VILIP-1* exon was identified as the potential promoter using the FirstEF (First Exon Finder) [16], Promoter scan [17] and Promoter 2.0 [18] programs. By aligning the human proximal 2-kb sequence with those of mouse and rat using the phastCons program [19], we found strong homology among these species, further indicating that this 2 kb sequence is conserved in different species and most likely corresponds to the human *VILIP-1* promoter. Due to the lack of a canonical TATA box, the *VILIP-1* promoter is numbered relative to the start of the first exon. Comparison of the 2 kb promoter sequences obtained from cell lines with that from database showed five polymorphisms at positions −1766, −1449, −1047, −893 and −324 (Table S2). However, these polymorphisms did not correlate with VILIP-1 expression levels. For example, polymorphisms at −1766 and −324 were observed in VILIP-1 expressing NCI-H520 cells and they were also detected in NCI-H522 and A549 cells in which *VILIP-1* was silenced.

### *In vitro* methylation represses *VILIP-1* promoter activity

Hypermethylation in CpG-rich promoters is strongly associated with transcriptional silencing [20]. In many types of cancer, a number of tumor suppressor genes are inactivated by promoter hypermethylation of CpG islands. Using the criteria formulated by Gardiner-Garden *et al*. [21], two *VILIP-1* promoter segments were identified to fulfill the strict definition of a CpG island, i.e. a 200-bp or greater stretch of DNA with a C/G content of >50% and an observed CpG/expected CpG ratio in excess of 0.6. The first 262-bp and second 434-bp regions (hereafter referred to as first and second CpG islands.) showed a CpG percentage of 68.3% and 63.4% respectively, and an observed/expected CpG ratio of 1.15 and 0.78 respectively. In order to determine whether the *VILIP-1* promoter activity is regulated by CpG methylation, we measured *VILIP-1* promoter activity under methylation and non-methylation conditions. To assay the effect of methylation on the promoter alone, we separately methylated the promoter fragment with M. SssI methylase, religated this fragment into the unmethylated plasmid, and transfected NCI-H520 cells and NCI-H522 cells with this construct. The methylation of *VILIP-1* promoter almost completely abrogated its activity (reduced from 100% to 10.4% for NCI-H520 and to 11.4% for NCI-H522), demonstrating that promoter methylation is sufficient for *VILIP-1* silencing (Figure 2G).

### Hypermethylation of *VILIP-1* promoter results in abrogation of VILIP-1 expression

**Figure 3:**
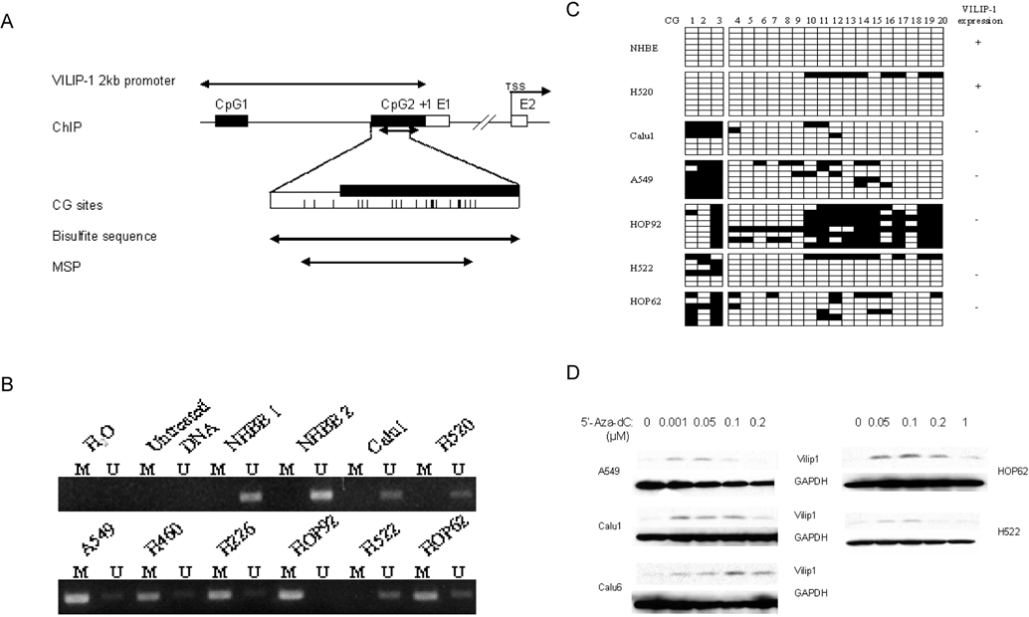
Methylation status of VILIP-1 promoter in NSCLC cell lines. A. Schematic map of VILIP-1 2kb promoter and CpG islands around exon1. Filled boxes, CpG islands. Open boxes, exons. Vertical ticks, CpG sites on the expanded axis. Start of exon 1 is marked at +1. TSS, translation start site (ATG start codon). B. MSP analysis of cell lines. Bands in lanes M are methylated, bands in lanes U are unmethylated. H_2_O: water was added instead of DNA; Untreated DNA: genomic DNA without treatment with sodium bisulfite; NHBE 1 and NHBE 2: DNA from two different individuals. C. Representative results of bisulfite sequencing of the second CpG island of VILIP-1 promoter in VILIP-1-expressing cell lines (+) and VILIP-1-nonexpressing cell lines (−). Open and filled squares indicate unmethylated and methylated CpG sites, respectively. Six to eight clones of PCR products amplified from bisulfite-treated genomic DNA were sequenced for each cell line. D. Reactivation of VILIP-1 expression by 5′-Aza-dC treatment in cell lines. VILIP-1 protein expression was determined by immunoblot analysis. GAPDH was included as a control for equal loading.

To define the methylation status of *VILIP-1* promoter, we designed a pair of methylation-specific primers and non-methylation-specific primers targeting 6 of the 20 CpG sites on the second CpG island for MSP (Figure 3A). The sequence of forward and reverse methylation-specific primers covered the CpGs 1 and 2 and CpGs 15-18, respectively. No methylation was detected in the promoter of normal primary cultures expressing VILIP-1 protein (NHBE1 and NHBE2). The promoter of the VILIP-1 expressing lung cancer cell line NCI-H520, was found to be minimally methylated by bisulfite sequencing (1 clone out of eight was found to be methylated) (Figure 3B and 3C). Conversely, the remaining NSCLC cells (A549, NCI-H460, NCI-H226, HOP62 and HOP92) displayed hypermethylation of the *VILIP-1* promoter. Very weak methylation was observed in NCI-H522 and Calu1. We further confirmed the MSP results by bisulfite sequencing. Figure 3C shows the methylation pattern of 20 CpG sites on the second CpG island. The methylation levels in all VILIP-1 non-expressing SCLC were higher than those in NHBE and NCI-H520 cells. Thus, these data reveal an inverse correlation between the methylation status of *VILIP-1* promoter and the respective gene expression in NSCLC cells.

### *Activation* of VILIP-1 expression in lung cancer cell lines after treatment with 5′-Aza-dC

To determine whether the absence of VILIP-1 expression in NSCLC cells, which have high degree of DNA methylation in the proximal 2 kb *VILIP-1* promoter, could be changed, we treated cells with 5′-Aza-dC for 5 days and evaluated the expression of VILIP-1 (Figure 3D). 5′-Aza-dC, a DNA methyltransferase inhibitor, at a concentration as low as 0.001 µM, restored VILIP-1 expression. Although the optimal concentration for activating *VILIP-1* expression was not always the same for all cell lines, a concentration of 0.1 µM 5′-Aza-dC had maximal induction effect on most cells, i.e., Hop62, NCI-H522, Calu-6 and Calu-1, whereas A549 cells only required 0.05 µM.

### Histone acetylation affects *VILIP-1* promoter activity

**Figure 4:**
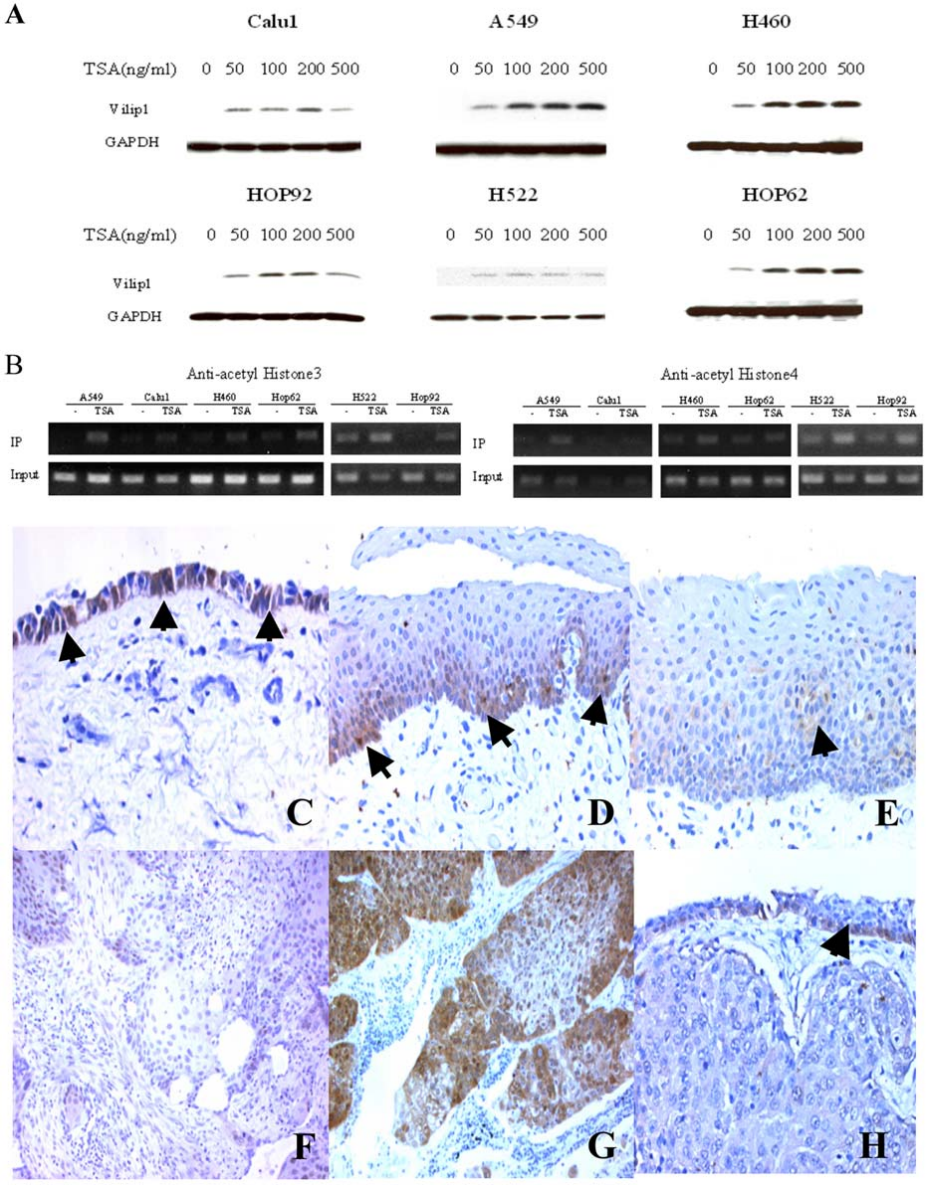
Modulation of VILIP-1 expression by histone acetylation. A. Induction of VILIP-1 expression by treatment with histone deacetylation inhibitor TSA in 6 cancer cell lines. Cells were treated for 20 hr with TSA at concentrations of 0, 50, 100, 200 and 500 ng/ml before lysis. VILIP-1 expression was detected by Western blot. GAPDH was used as loading control. B. Effect of TSA treatment on the acetylation status of histones H3 and H4 at the second CpG island of *VILIP-1* promoter in A549, Calu1, NCI-H460, HOP62, H522 and HOP92 cells. Cells were treated with TSA (200 ng/ml) for 20 hr and histone acetylation status was analyzed using chromatin immunoprecipitation. Amplification of DNA before precipitation (1% of the total sample) was used as input. VILIP-1 immunohistochemistry of bronchial epithelium showing positively stained cells (arrows): Normal mucosa (C), regular metaplasia exhibiting positive immunostain in basal and parabasal layers (D), metaplasia with dysplasia has little to no VILIP-1 expression (E). Panel F shows a SCC without VILIP-1 expression, whereas in panel G the protein is expressed abundantly in approximately 50% of tumor cells. Adenocarcinoma (H) shows no VILIP-1 immunostain, note that the covering bronchial epithelium in this panel shows a few positive basal cells (arrow). ×100.

A growing body of data indicates that gene silencing is also modulated by histone deacetylation, an epigenetic mechanism different from methylation [22]. Therefore, we also investigated the potential role of histone acetylation in the regulation of VILIP-1 expression by treating cells with the histone deacetylase inhibitor, TSA. TSA potently reactivated VILIP-1 expression in Calu1, A549, NCI-H460, HOP92, NCI-H522 and HOP62, at an optimal concentration of approximately 200 ng/ml (Figure 4A). To assess the relationship between the degree of histone acetylation and VILIP-1 expression, we performed ChIP assays using antibodies against acetylated histone H3 (at lysines 9 and 14) and H4 (at lysines 5, 8, 12 and 16). After amplification with primers specific for the second CpG island, we observed TSA increased the acetylation of both histones H3 and H4 (to lesser extent) which interacted with the *VILIP-1* promoter (Figure 4B) in all six cell lines studied. Thus, the acetylation status of histones H3 and H4 correlated with the expression of VILIP-1.

### Expression of *VILIP-1* in primary lung tumors and survival

VILIP-1 immunohistochemistry was performed on TMA sections to assess its expression in NSCLC. In addition, we used 21 conventional paraffin blocks to evaluate normal pulmonary tissue and precursor bronchial lesions.

**Figure 5:**
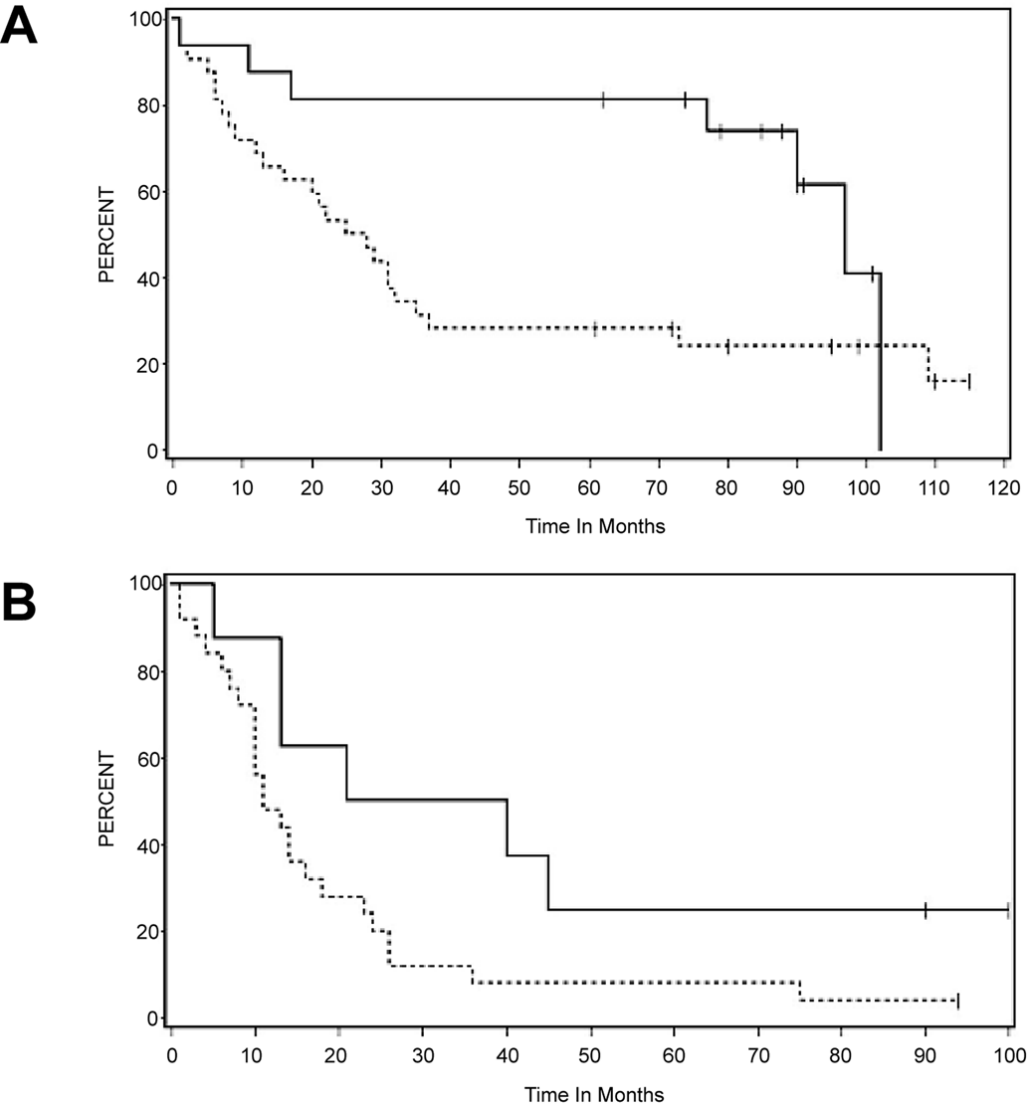
Kaplan Meir curves for VILIP1 groupings. Dotted line: Low expression (IHC score 1–4), full line: High expression (IHC score 5–8). A: Early clinical stages (stages 1 or 2) and B: Late stages (stage 3 or 4).

Normal bronchial mucociliary epithelium expressed VILIP-1 in all cases. This expression was mostly limited to the basal layer, where the intensity was moderate to intense and encompassed 50–100% of basal cells (Figure 4C). In 10 hyperplastic and metaplastic epithelia, the immunostain appeared mostly in the basal and parabasal cells (Figure 4D). Five out of nine moderate and severe dysplasias expressed little or no VILIP-1 (Figure 4E). VILIP-1 was not expressed in approximately 25% invasive SCCs (Figure 4F). Nevertheless, 44% of SCCs had scores between five and eight (moderate-high expression) (Figure 4G) and 31% exhibited scores between one and four, representing marginal to mild expression. Adenocarcinomas showed a different pattern, i.e., the majority (86%) showed no immunostain at all (Figure 4H) and 14% of these tumors expressed VILIP-1. Only 8% showed high levels of expression. VILIP-1 expression in NSCLC patients (SCC plus adenocarcinoma cases, n = 81) surviving for more than 5 years was significantly higher than in those patients that survived for less than 5 years (p<0.0001) Further, after adjusting for tumor stage (p<0.006), grade (p<0.400) and histology (p<0.032), VILIP1 remained a significant predictor of time to death (p<0.006). Figure 5 displays Kaplan-Meier plots for VILIP1 groupings (low versus high expressors), separately for early stage (stages 1 or 2) and late stage (stage 3 or 4) tumors. In early stage disease, median survivals in the low and high VILIP1 groups were 26 and 97 months, respectively. In later stage disease, median survivals in the low and high VILIP1 groups were 11 and 30.5 months, respectively.

A statistically significant level was also seen when patients were stratified in either SCC (n = 36) or adenocarcinoma subgroups (n = 45), (p<0.025). Although there was a tendency to see low levels of VILIP-1 expression in patients with high clinical stages, no statistically significant difference could be demonstrated.

### *VILIP-1* expression and promoter methylation in primary lung tumors

We further interrogated the correlation between promoter methylation and expression of VILIP-1 in 21 primary human NSCLC. We studied the VILIP-1 promoter methylation using MSP. Four of 5 SCC tissues with reduced VILIP-1 expression showed methylation in the VILIP-1 promoter and the other SCC had no methylation (see supplemental Figure S1). No or very weak methylation was detected in 3 of 6 SCC with high VILIP-1 expression. In adenocarcinomas, 8 of 10 tissues displayed methylation. Since most adenocarcinoma did not express VILIP-1, we focused our attention on SCCs. In order to examine the clinical significance of VILIP-1 expression in SCC patients, we selected 56 SCC samples and categorizing them into two groups (short survival, less than 2 years and long survival, more than 5 years). These specimens were evaluated for VILIP-1 protein expression using IHC, and promoter methylation using MSP. We detected no or weak VILIP-1 signal in one third of SCCs. The patients with longer-than-5-year overall survival had significantly higher VILIP-1 expression than those with shorter-than-2-year survival (*P*-value<0.007).

**Figure 6:**
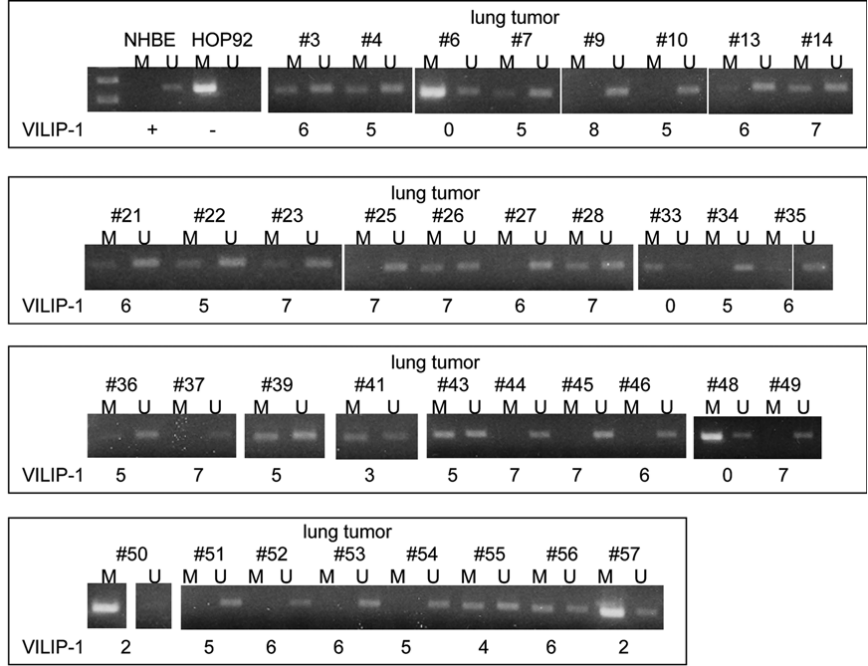
MSP analysis of representative primary lung SCC. Bands in lanes M are methylated, bands in lanes U are unmethylated. NHBE and HOP92 cells were used as controls. VILIP-1 expression is indicated under each case, using the IHC score.

SCC samples with low level of VILIP-1 expression (IHC score of 0–4) were significantly (p<0.05) more likely to exhibiting VILIP-1 promoter methylation than samples with high VILIP-1 expression (score>4). The methylation rates were 71% and 41% for low and high VILIP-1 expression groups, respectively (Figure 6).

## Discussion

Both genetic and epigenetic abnormalities contribute to lung carcinogenesis. Activation of *K-ras* and inactivation of *p53*, *Rb*, and *p16*, were identified as the predominant alterations in lung cancer [7], [23], [24]. A number of genes regulating many cellular functions such as cell cycle, DNA repair, Ras signaling, invasion, etc are inactivated by promoter hypermethylation in lung cancer [8], [22], [24], [26], [28], [29]. In the present study, we found that expression of VILIP-1 was frequently lost in human lung cancer cells and that silencing of its expression was due to epigenetic changes.

VILIP-1 has been implicated in regulating cell signaling during development and differentiation in the central nervous system [1], [2]. VILIP-1 is also widely expressed in sites outside the nervous system such as human heart, lung, liver and testis and moderately expressed in ovary, kidney, spleen and pancreas, suggesting that VILIP-1 might be required for the maintenance of tissue homeostasis in different organs [25]. Given the central role of VILIP-1 as a calcium sensor in mediating cAMP response, deregulation of VILIP-1 expression may cause malfunction in multiple organ systems. Indeed, recent studies including VILIP-1 downregulation in murine skin and human esophageal squamous cell carcinomas support this view [3], [4], [5]. Using the NCI-60 panel of cancer cells, we found that VILIP-1 protein was expressed in cancer cells from the central nervous system and colon, while it appeared to be undetectable in melanoma and cancers of the lung, breast, ovary, and kidney. Since VILIP-1 expression was detected in normal tissues from lung, ovary and kidney [25], VILIP-1 is down-regulated in cancer cells derived from these tissues. We confirmed that VILIP-1 is silenced in most NSCLC cells by comparing a dozen tumor cell lines with normal human bronchial epithelial cells and investigated the mechanisms underlying VILIP-1 down-regulation. In this context, we did not find significant mutations in any of the 4 exons of the *VILIP-1*. In addition, neither the exon-intron junctions nor the *VILIP-1* proximal 2kb promoter showed any alterations. Since genetic alterations were not responsible for *VILIP-1* silencing in NSCLC, we embarked in assessing possible epigenetic mechanisms of *VILIP-1* silencing and identified both promoter hypermethylation and histone modifications.

In this study, we demonstrated that methylation of the CpG island within the *VILIP-1* promoter was a significant mechanism mediating *VILIP-1* silencing in NSCLC: Methylation-induced suppression of gene transcription may occur by direct interference with the binding of transcription factors such as Sp-1/Sp-3 [20], [26]. To our knowledge, the promoter of *VILIP-1* has not previously been reported. Among the family of neuronal calcium sensor proteins related to VILIP-1, only the promoter of human KChIP4 was predicted to contain four Sp-1-binding elements [27]. Analysis of the *VILIP-1* promoter revealed multiple potential Sp-1 binding sites within both CpG islands (data not shown). Thus, it is possible that methylation of the *VILIP-1* promoter mediates gene silencing by blocking the binding of Sp-1 and other transcription factors to the *VILIP-1* promoter.

Another epigenetic regulatory mechanism in human cancer is related to the inactivation of tumor suppressor genes through the post-transcriptional modification of the N-terminal histone tails that protrude from the nucleosome core [20], [28], [29]. The promoters of silenced genes contain localized regions of transcriptional-silencing marks that include the deacetylation of lysines 9 and 14 and the methylation of lysine 9 of histone H3. Transcriptional-activation marks such as hyperacetylation of histones H3 and H4 and methylation of lysine 4 at H3 allow gene transcription. These marks form the histone code [28]. Acetylation and deacetylation of histones by histone acetyltransferases and histone deacetylases (HDACs) alter chromatin structure in a way which dynamically affects transcriptional regulation [30], [31]. Inhibition of HDACs by HDAC inhibitor causes accumulation of hyperacetylated histones and acetylation of transcription factors, leading to transcriptional activation of genes involved in cancer cell growth, apoptosis, differentiation, migration and invasion. Accumulating data show that one of these HDAC inhibitors, TSA, can cause the reactivation of a number of tumor suppressor genes such as TGF-beta receptor type II [32], death-associated protein kinase [33], CCAAT/enhancer-binding protein α [34] and MYO18B [35] in lung cancer. Re-expression of tumor suppressor genes via induced acetylation of histones H3 and H4 by TSA could induce apoptotic cell death in human lung cancer cells [36]. Interestingly, Zhong et al [37] recently used expression profiling to analyze novel targets for epigenetic modification in human lung cancer and revealed that silencing by histone deacetylation was nearly as common as silencing by DNA methylation in a panel of nine genes. Five tumor suppressors or suppressor candidates including NRIP3, CYLD, CD9, ATF3 and OXTR were strongly induced by TSA alone. In the present study we found that TSA treatment with concentrations ranging from 50 to 500 ng/ml potently reactivated VILIP-1 expression in all the lung cancer cell lines tested. Further analysis of the TSA action mechanism indicated that TSA enhanced the binding of acetylated histones H3 and H4 at the *VILIP-1* promoter, therefore reaching the transcriptional-activation mark of the histone code. Acetylated lysines could recruit the chromatin remodeling complex SWI/SNF which in turn, via its ATPase activity, displaces and twists nucleosome exposing VILIP-1 promoter for interaction with the transcription machinery [22].

A percentage of early bronchial precursor lesions show decreased levels of VILIP-1 expression, indicating that this protein may be starting to decrease early during carcinogenesis. Additional studies with larger numbers of *in situ* lesions will be required to confirm this impression. The study of primary lung tumor specimens showed clearly that a statistically significant difference in survival was associated with VILIP-1 expression. High levels of VILIP-1 expression were seen in NSCLC patients that had a longer survival whereas, absent or low levels of expression were seen in patients with poorer outcomes. This relationship was assessed in the specimens studied by immunohistochemistry either in TMAs or in regular paraffin block sections. Furthermore, this significant difference was evident not only in the entire group of NSCLC patients but also when the population was further stratified into SCC and adenocarcinoma patient subgroups. Irrespective of histological type, VILIP-1 expression was significantly reduced in more advanced stages of NSCLC than in stages 1–2, another indication that VILIP-1 silencing is associated to tumor progression. Overall, the statistical analysis of the data showed that VILIP-1 is a promising prognostic outcome predictor that could be used in the clinic.

In conclusion, we found that the VILIP-1 is down-regulated in the most common human lung cancer histotypes. Decreased expression of VILIP-1 was associated with poorer outcomes in the NSCLC patients that showed a statistically significant reduction in survival. Epigenetic regulations including promoter hypermethylation and histone modification rather than genetic alterations are responsible for VILIP-1 silencing.

## Supporting Information

Table S1 (5.44 MB TIF)

Table S2 (0.38 MB TIF)

Figure S1 MSP analysis of representative primary lung adenocarcinomas and SCCs. Bands in lanes M are methylated, bands in lanes U are unmethylated. NHBE and HOP92 cells were used as controls.
(1.84 MB TIF)
