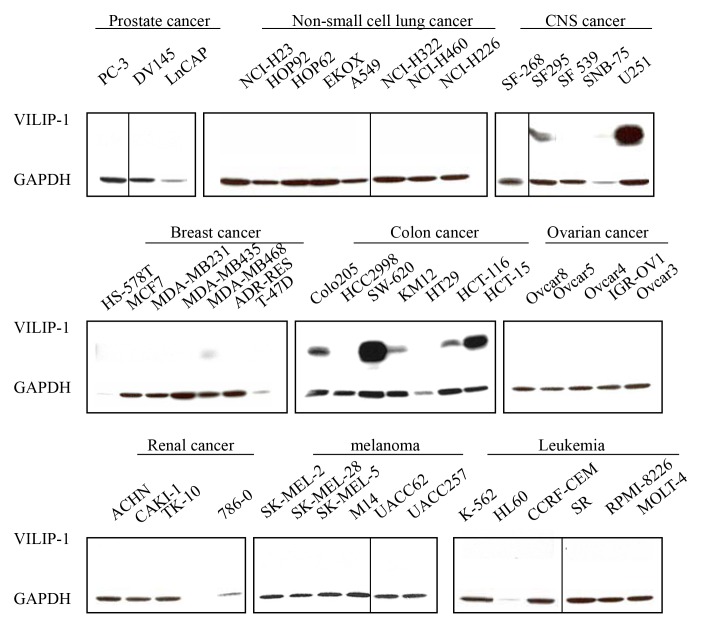# Correction: *VILIP-1* Downregulation in Non-Small Cell Lung Carcinomas: Mechanisms and Prediction of Survival

**DOI:** 10.1371/annotation/ad0e4775-c4d5-454c-96c0-755ba9fcc806

**Published:** 2013-10-24

**Authors:** Jian Fu, Kathryn Fong, Alfonso Bellacosa, Eric Ross, Sinoula Apostolou, Daniel E. Bassi, Fang Jin, Jirong Zhang, Paul Cairns, Inmaculada Ibañez de Caceres, Karl-Heinz Braunewell, Andres J. Klein-Szanto

The authors would like to provide a clarification in relation to Figure 1 in the article.

Although all the cell lines/lanes were exposed together (see accompanying original raw blots), most site panels were not run in adjacent lanes but were cut and pasted to fit the figure format. We provide a revised Figure 1 where a dividing vertical line indicates which lanes were not adjacent in the raw blot. Furthermore, the bands for the breast cancer panel are slightly overcropped and a band for the CNS panel (present in the original raw blot) was cut off. In part, this is due to the fact that cell line SF295 was ran together with the positive control lung cell line H-520 (see figure 2) that is not a member of the NIH-60 cell panel and was cropped out from the original raw blot to fit the description of this figure dedicated to the NCI-60 cell panel.

Raw Blots: 


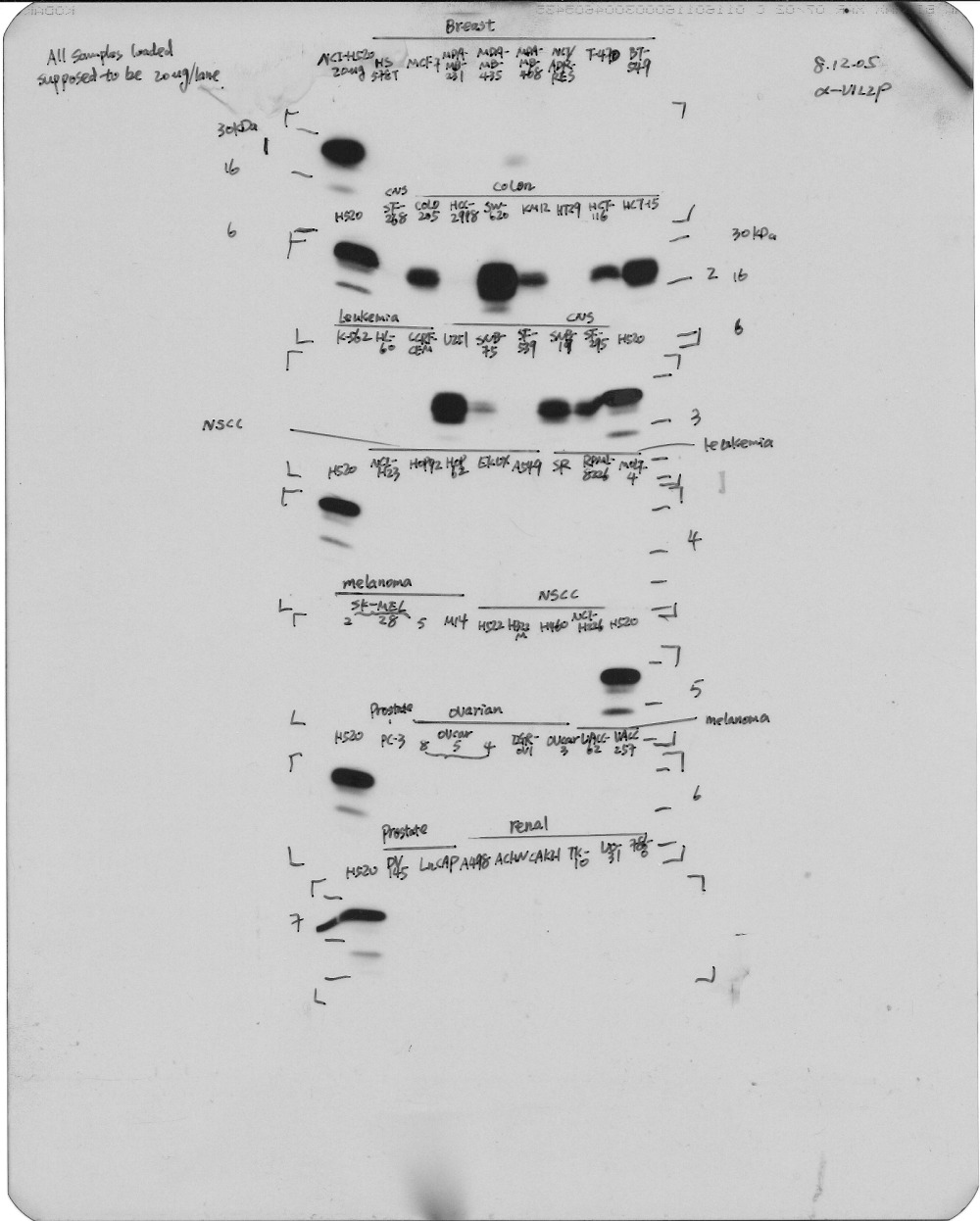
(1)

Figure 1: 

**Figure:**