# Supplementary figures and images for: VILIP-1 Downregulation in Non-Small Cell Lung Carcinomas: Mechanisms and Prediction of Survival
Source: PLoS One. 2008 Feb 27;3(2):e1698. doi: 10.1371/journal.pone.0001698 (PMC2246032; doi:10.1371/journal.pone.0001698)

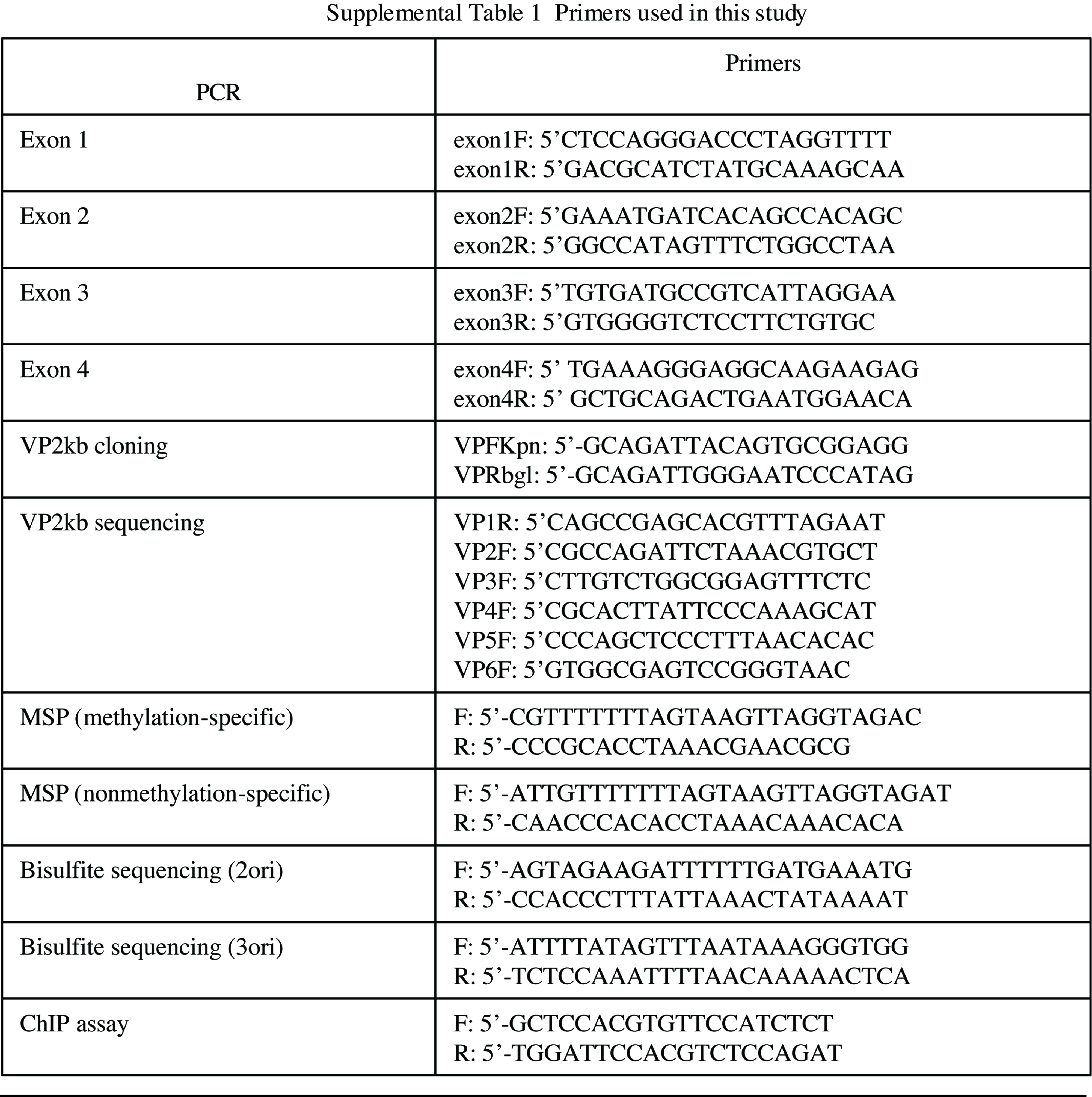

Supplement: Table S1 — (5.44 MB TIF) [file pone.0001698.s001.tif]

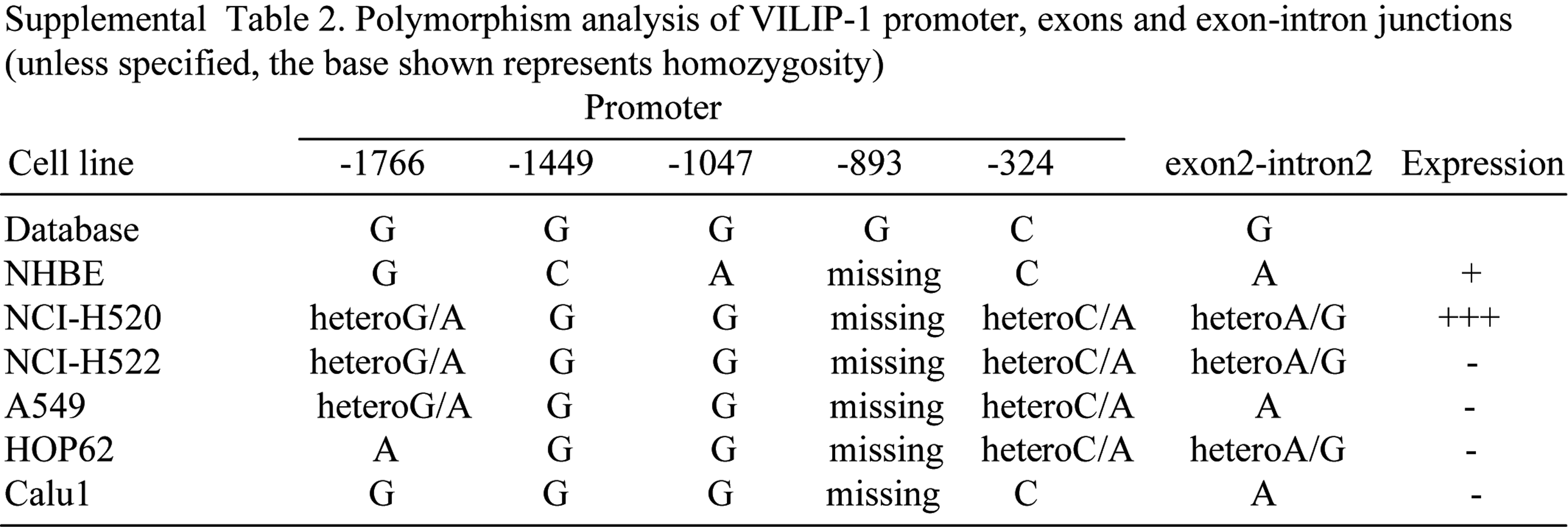

Supplement: Table S2 — (0.38 MB TIF) [file pone.0001698.s002.tif]

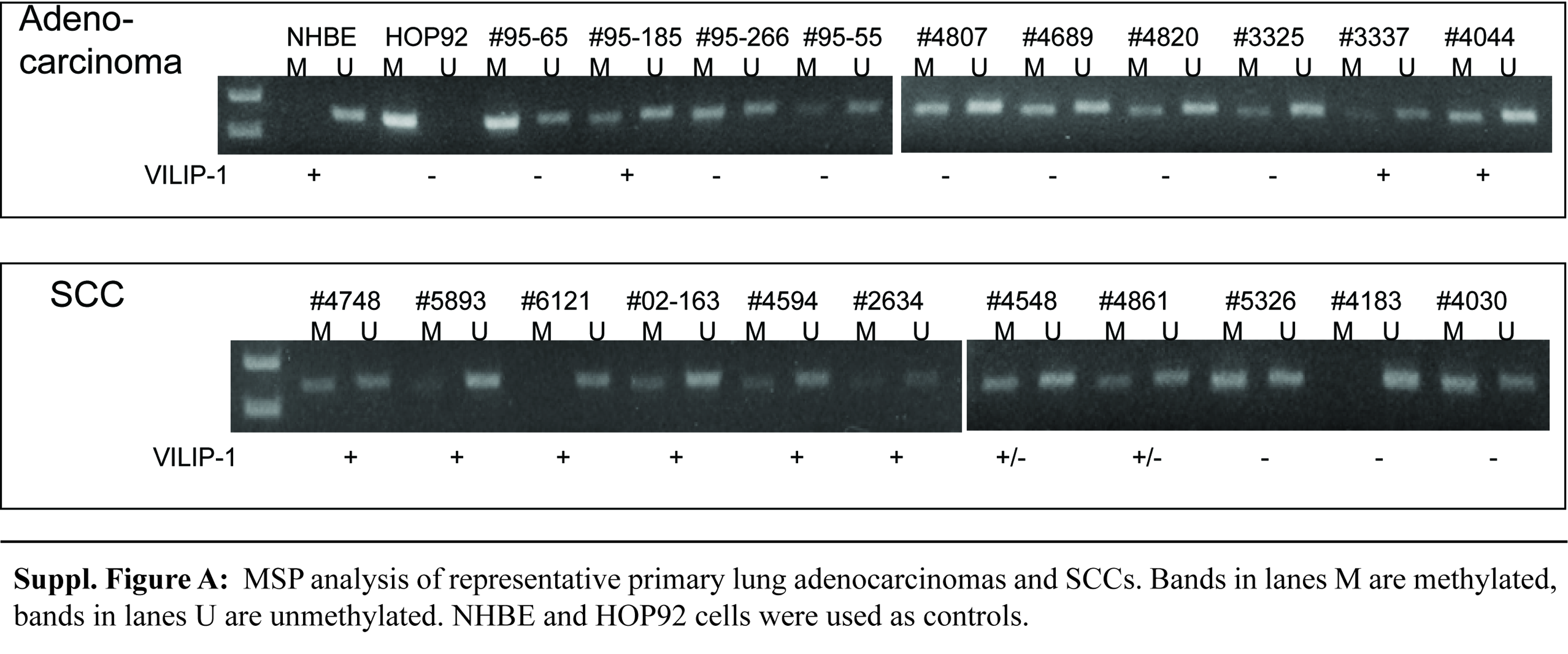

Supplement: Figure S1 — MSP analysis of representative primary lung adenocarcinomas and SCCs. Bands in lanes M are methylated, bands in lanes U are unmethylated. NHBE and HOP92 cells were used as controls. (1.84 MB TIF) [file pone.0001698.s003.tif]
